# Eye Care Quality and Accessibility Improvement in the Community (EQUALITY) for adults at risk for glaucoma: study rationale and design

**DOI:** 10.1186/s12939-015-0213-8

**Published:** 2015-11-18

**Authors:** Cynthia Owsley, Lindsay A. Rhodes, Gerald McGwin, Stephen T. Mennemeyer, Mary Bregantini, Nita Patel, Demond M. Wiley, Frank LaRussa, Dan Box, Jinan Saaddine, John E. Crews, Christopher A. Girkin

**Affiliations:** Department of Ophthalmology, School of Medicine, University of Alabama at Birmingham, 700 S. 18th St, Birmingham, AL 35294-0009 USA; Department of Epidemiology, School of Public Health, University of Alabama at Birmingham, 1655 University Blvd, Birmingham, AL 35294-0022 USA; Department of Health Care Organization and Policy, School of Public Health, 1655 University Blvd, Birmingham, 35294-0022 USA; Prevent Blindness, 211 West Wacker Drive, Suite 1700, Chicago, Il 60606 USA; Walmart Vision Center #1481, Birmingham, AL 35209 USA; Walmart Vision Center #715 and Global Eye Care PC, Tuscaloosa, AL 35405 USA; Vision Health Initiative, Division of Diabetes Translation, Centers for Disease Control and Prevention, Atlanta, GA 30341-3727 USA

**Keywords:** Primary open angle glaucoma, Ocular hypertension, Glaucoma suspect, Health disparities, Telemedicine, Vision impairment, Eye care utilization, Eye health education, Spectral domain optical coherence tomography

## Abstract

**Background:**

Primary open angle glaucoma is a chronic, progressive eye disease that is the leading cause of blindness among African Americans. Glaucoma progresses more rapidly and appears about 10 years earlier in African Americans as compared to whites. African Americans are also less likely to receive comprehensive eye care when glaucoma could be detected before irreversible blindness. Screening and follow-up protocols for managing glaucoma recommended by eye-care professional organizations are often not followed by primary eye-care providers, both ophthalmologists and optometrists. There is a pressing need to improve both the accessibility and quality of glaucoma care for African Americans. Telemedicine may be an effective solution for improving management and diagnosis of glaucoma because it depends on ocular imaging and tests that can be electronically transmitted to remote reading centers where tertiary care specialists can examine the results. We describe the Eye Care Quality and Accessibility Improvement in the Community project (EQUALITY), set to evaluate a teleglaucoma program deployed in retail-based primary eye care practices serving communities with a large percentage of African Americans.

**Methods/Design:**

We conducted an observational, 1-year prospective study based in two Walmart Vision Centers in Alabama staffed by primary care optometrists. EQUALITY focuses on new or existing adult patients who are at-risk for glaucoma or already diagnosed with glaucoma. Patients receive dilated comprehensive examinations and diagnostic testing for glaucoma, followed by the optometrist’s diagnosis and a preliminary management plan. Results are transmitted to a glaucoma reading center where ophthalmologists who completed fellowship training in glaucoma review results and provide feedback to the optometrist, who manages the care of the patient. Patients also receive eye health education about glaucoma and comprehensive eye care. Research questions include diagnostic and management agreement between providers, the impact of eye health education on patients’ knowledge and adherence to follow-up and medication, patient satisfaction, program cost-effectiveness, and EQUALITY’s impact on Walmart pharmacy prescription rates.

**Discussion:**

As eye-care delivery systems in the US strive to improve quality while reducing costs, telemedicine programs including teleglaucoma initiatives such as EQUALITY could contribute toward reaching this goal, particularly among underserved populations at-risk for chronic blinding diseases.

## Background

Primary open angle glaucoma (POAG) is a chronic, progressive optic neuropathy characterized by changes in the optic disk, thinning of the retinal nerve fiber layer, and gradual loss of vision beginning in the peripheral field and extending to central vision in advanced disease. The prevalence of POAG increases dramatically with age, affecting more than 1.8 % of the US population over 40 but increasing to 23.2 % among African Americans and 9.4 % among Whites over the age of 75 [1]. POAG is 4–5 times higher in African Americans as compared to Americans of European descent [2, 3]. In addition, the disease progresses more rapidly and appears about 10 years earlier in African Americans [4–10]. Given the rapid expected growth in older populations, the number of POAG cases will increase in the US by 50 % by 2020, directly effecting 3.36 million lives [1]. Given projected Medicare shortfalls and potential reductions in Medicaid coverage in the US [11], developing high-quality, cost-effective access to eye care for these individuals may become increasingly important.

The personal burden of POAG and accompanying vision impairment faced by individuals with this condition is significant and has been widely documented. Adults with POAG experience reductions in health-related quality of life and mobility problems such as ambulatory and driving difficulties [12–18]. Persons with vision impairment, regardless of etiology, are at increased risk for depression, social disengagement, employment challenges, and problems accessing health care [19–23]. Mortality risk is increased for persons with vision impairment [24, 25], including for those with POAG [26, 27]. Cost-effective benefits to quality of life have been reported for early detection and treatment of POAG [28–32]. Thus, public health efforts to implement interventions for early detection and effective follow-up management of POAG are likely to improve patients’ health and well-being.

Older African Americans are less likely to receive routine, comprehensive eye care as compared to whites, during which POAG could be detected and treated in a timely fashion [33–35], and they tend to receive fewer health care services [36]. This lack of care may contribute to their higher rates of POAG and associated vision impairment. When this population enters treatment, their POAG is often in more advanced forms accompanied by irreversible vision impairment, and thus more difficult to treat, as compared to whites. Further, socioeconomic disparities experienced by the African American population may limit their ability to receive follow-up eye care after diagnosis—a crucial factor for slowing disease progression.

Research has demonstrated that inadequate knowledge and awareness about the importance of eye care and strategies for minimizing barriers to care contribute to African Americans’ compliance problems with eye disease management plans and their low eye care utilization rate [37, 38, 23, 39–41]. These factors include inadequate knowledge about basic symptoms, risk factors and treatments for commons eye diseases and conditions including glaucoma; lack of awareness about the importance of routine preventive care; cost for exams, co-pays, and spectacles; transportation challenges; and communication and trust issues with the doctor and staff. Over the past decade progress has been made in developing eye health education materials and interventions that have efficacy in improving their eye health knowledge and in facilitating eye care seeking behaviors. These eye health education programs are typically focused on improving knowledge and attitudes about eye health, disease and prevention, and communicating strategies to remove the perceived barriers to care [42–45]. Two clinic-based studies involving inner-city African-Americans with diabetes who were not receiving annual eye care showed that the dilated fundus exam rate increased after distribution of educational material and telephone follow-up emphasizing the importance of annual eye care [46–48]. A multimedia campaign in California combined with patient interaction increased the use of eye care services [49]. Here in Alabama we have shown that an eye health educational presentation whose content was targeted specifically for African Americans successfully imparted information about the importance of annual preventive care, which was retained 3–6 months later [44, 40, 50]. Thus, there is evidence that eye health educational interventions can have positive impacts on knowledge, attitudes, and beliefs about eye care and on eye health behaviors.

There are other factors that contribute to later diagnosis of POAG and under-utilization of eye care in African Americans. The number of patients requiring ophthalmic care is expected to increase by 18.1 % between 2008 and 2015, whereas the number of US ophthalmologists is expected to grow only 0.67 % in the same time period [51, 52]. The Affordable Care Act in the US was designed to increase the number of Americans with health insurance, reducing some financial barriers to glaucoma detection and treatment. However, this may also increase the demand for scarce clinical resources. Furthermore, ophthalmology practices are rarely located in rural counties of southern states where African Americans represent the majority of the population [53], thus creating further problems related to accessing ophthalmological care, including care provided by fellowship-trained glaucoma specialists.

There is considerable evidence [54–60] that the recommended screening and follow-up protocols for managing glaucoma from the American Academy of Ophthalmology Preferred Practice Patterns (PPP) [61], which are very similar to the practice guidelines recommended by the American Optometric Association [62], are often not followed by primary eye-care providers, including both ophthalmologists and optometrists. This situation is unfortunate because optimal management of open angle glaucoma depends upon careful interval examination and documentation of ophthalmic findings with specific emphasis on monitoring the structure and function of the optic nerve in order to diagnosis the condition or to detect progressive injury [63]. Chart review studies in the US [64, 56, 58] document deficits in current care delivery compared to the PPP. While intraocular pressure (IOP) is frequently recorded, the primary areas of weakness are in the performance and/or documentation of gonioscopy, the documentation of optic nerve appearance, and the frequency of visual field testing [65, 56, 58, 64]. Although patients overall are likely to be scheduled for follow-up within PPP-recommended intervals, patients with unstable POAG, who are at higher risk of visual loss compared to those with stable POAG, are more likely to fall outside these intervals [56]. Administrative database studies in the US confirm these findings showing a large variation in practice patterns, with frequent deficiencies in gonioscopy [54, 55], recording of optic nerve structure [56, 58], visual field testing [56, 66] and frequency of follow-up visits [57]. Of note, African Americans were less likely to receive appropriate pre-operative testing as compared to whites [66]. Thus the literature strongly supports the existence of deficiencies in glaucoma management likely to dramatically reduce the ability of clinicians to detect potentially blinding progressive glaucomatous injury. It is likely that the problem is significantly worse in at-risk minority populations, such as African American individuals. Challenges in the accessibility and quality of glaucoma care have not been adequately addressed in public health initiatives and is a critical need if more effective healthcare delivery programs are to be developed in underserved settings.

Taken together, these factors suggest the consideration of an alternative model of glaucoma care in US communities with high percentages of persons at-risk for POAG, including African Americans. The use of telemedicine could conceivably be a key part of such a model. Telemedicine refers to the electronic communication of medical information from one site to another to improve a patient’s clinical health status [67]. It has been placed into practice in many healthcare fields and can increase patient satisfaction and accessibility to specialty care. By utilizing current technologies, telemedicine transmits patient data from a primary eye care clinic to another remote site for review by physicians specializing in the conditions being assessed (in the case of “teleglaucoma” programs, ophthalmologists fellowship-trained in glaucoma). The patient does not need to be seen in person by the specialist, thus making the evidence-based standards of specialty care more accessible to patients living in geographically remote areas with no specialty practice or to those with transportation challenges. The vast majority of the literature on telemedicine in ophthalmology is focused on diabetic retinopathy screening since there have been great strides in the development of non-invasive retinal imaging devices whose results can be electronically transmitted. These tests provide high levels of diagnostic reliability and ease of training of testing personnel [68–70]. Acceptance of telemedicine for diabetic retinopathy has increased steadily around the world over the past 10 years stemming from its proven efficacy and cost-effectiveness [71–74]. The detection and management of glaucoma also heavily relies on ocular imaging and other specialized tests whose results can be electronically transmitted. However, teleglaucoma programs have lagged somewhat behind diabetic retinopathy telemedicine programs in their development and implementation. In the past few years, there have been a handful of articles describing pilot teleglaucoma programs in various parts of the world (e.g., US, UK, Canada, Finland, Kenya, Greece, Australia) [74–84]. This literature has largely focused on emphasizing the public health need for telemedicine services for glaucoma in rural and outlying areas, demonstrating feasibility, and the importance of multi-disciplinary collaboration among types of providers. In addition, previous attempts to use telemedicine in glaucoma care primarily utilized transmission of optic disc photos, which can give rise to problems such as inadequate quality of transmitted stereoscopic images, variations among glaucoma specialists’ subjective evaluations of images, and the high false-positive rate of some optic nerve imaging modalities [85, 78, 81, 86–93].

Owing to these considerations, we designed and implemented a glaucoma detection and management program consisting of a community-based teleglaucoma model particularly targeted at the at-risk African American population ≥40 years old. The program is entitled Eye Care Quality and Accessibility Improvement in the Community (EQUALITY), and is described in detail in Methods. In order to evaluate this program, the following questions will be addressed: (1) What is the impact of patient education about glaucoma and the importance of routine comprehensive eye care on a patients’ knowledge about glaucoma-associated diagnoses (GAD), including glaucoma, glaucoma suspect, and ocular hypertension, adherence with recommended follow-up examinations for GAD, and adherence to filling medication prescriptions for GAD? (2) What is the level of patient satisfaction with teleglaucoma care based in the clinic in their community? (3) To what extent does the primary eye care provider follow the PPP in managing the care of those at-risk for glaucoma and those already diagnosed with glaucoma? (4) What is the extent of diagnostic agreement between the primary eye care provider and the ophthalmologist who has completed specialty fellowship training in glaucoma? (5) What is the cost-effectiveness of this telemedicine program compared to usual in-person care of a glaucoma patient by a glaucoma specialist? (6) What is the impact of the EQUALITY program in the primary eye care clinic in a large, national retailer on the rate of GAD prescriptions being filled in their pharmacy, as compared to the rate in a large national retailer with a primary care eye clinic where the EQUALITY program was not administered?

## Methods

The study design is an observational prospective examination of a model of glaucoma detection and management. All patients receive the same model of care. Patients at-risk for POAG (per criteria described later) are seen for routine dilated comprehensive eye care by primary eye-care providers, in this case optometrists, working within retailer-based clinics in two predominantly African American communities in Alabama (one urban/suburban and one rural). A telemedicine process is implemented whereby the results of the comprehensive examination as well as optic nerve imaging and functional testing are electronically transmitted to a server at a remote tertiary care glaucoma center staffed by an ophthalmologist who has completed fellowship training in glaucoma (hereafter called the glaucoma specialist). The results of the examination, including imaging and other procedures, are reviewed by the glaucoma specialist, who provides feedback to the optometrist, who then recommends a treatment plan to the patient. During the clinic visit, a trained technician delivers evidence-based eye health education and discusses the importance of routine eye care and adherence with doctor’s recommendations to enhance sight preservation. The advantages of this approach are three-fold in terms of reaching the long-term goal of improving the quality of and access to eye care for persons at risk for POAG. First, it allows for the remote application of specialist-level clinical evaluation to primary eye care clinics providing care to socioeconomically disadvantaged individuals who have significant barriers (e.g., travel distance) to receiving higher-level evaluations. Second, it provides eye health education messages specifically targeted to this at-risk population. Third, this telemedicine program can potentially be scaled and replicated nationally given the ubiquity of large, national retailers with primary care vision centers in the US.

The Institutional Review Board of the University of Alabama at Birmingham (UAB) reviewed and approved the study’s protocol. The geographical setting for the study consists of two regions in Alabama with a high percentage of African Americans. One setting is Homewood, Alabama, a community within the Birmingham, Alabama, metropolitan area, the largest city in the state. Birmingham’s population is approximately 73 % African American. The second setting is Tuscaloosa, Alabama, the county seat of Tuscaloosa County. Tuscaloosa County is located in west Alabama at the edge of Alabama’s Black Belt region, named for its rich black soil. The Black Belt is a rural area with one of the highest poverty rates in the U.S. and has been characterized as “Alabama’s Third World” [94]. It has among the highest concentrations of African American residents of any rural region of the country, representing over 50 % of the population. In addition to widespread poverty, the area is characterized by inadequate education, transportation, and community resources, as well as a shortage of healthcare providers.

The study settings are two primary eye care clinics in Homewood and Tuscaloosa, Alabama. The clinics are Walmart Vision Centers, located next to a large general retailer–a Walmart Supercenter. Each clinic is independently owned and operated by an optometrist who has an established practice at the Vision Center (10–15 years duration) with high patient volume (4000 patient visits per year in Homewood clinic and 7000 patient visits per for Tuscaloosa clinic). Fifty percent or more of patients seen in these clinics are African American. We selected Walmart Vision Centers as our primary eye care clinics for several reasons. In these communities, Walmart, a general retailer, is popular with community residents seeking groceries, household items, clothing, prescription and over-the-counter medications, and car care. Thus, this retailer has a high level of familiarity to residents of the region. Furthermore, Walmart has an extensive network of Vision Care Centers throughout the US, providing an infrastructure to replicate and scale up a telemedicine network. If the efficacy of the EQUALITY program is established, the intervention would be suitable for any large general retailer with primary eye care clinics.

Participants in the study are new or existing patients presenting for appointments at the clinic who meet any of the following criteria: (1) African Americans ≥40 years old, (2) Whites ≥50 years old, (3) persons of any age or race/ethnicity with diabetes, (4) persons of any age or race/ethnicity with a glaucoma associated diagnosis (GAD) (glaucoma suspect (GS), ocular hypertension (OHT), and POAG), (5) persons with a self-reported family history of POAG. These criteria were selected because they are established risk factors for glaucoma [61]. The enrollment period is from May 2013 through May 2014 for the Tuscaloosa, Alabama, Vision Center and May 2013 through July 2014 for the Homewood, Alabama, Vision Center. Patients are seen in the clinic Monday through Friday.

All patients who are eligible and enroll in the study receive a comprehensive eye examination (CEE) by the optometrist after their arrival at the clinic. The CEE consists of collection of demographic and other information (birthdate, gender, race/ethnicity, address of residence, health insurance status), patient history (chief complaint, history of presenting illness, ocular history, medical history, family and social history), blood pressure, ocular examination (visual acuity with walk-in and best correction, refraction, color vision, applanation tonometry, pachymetry, undilated slit lamp anterior segment examination, undilated gonioscopy, dilated fundus examination). After the CEE, the optometrist determines the patient’s ocular diagnosis and whether the patient has normal ocular results, has a GAD, or has other ocular disease. Table 1 provides information on the definition of normal and GADs [95]. All data from the CEE are placed in the electronic database viewable at the clinic site by the optometrist and by the glaucoma specialist at the UAB Glaucoma Service; this database also contains all subsequent structural and functional testing as described below. The protocols for the process evaluation for each of the possible diagnostic outcomes are outlined below.

**Table 1:** Case definitions for glaucoma and glaucomatous conditions [95]

	Definition
Normal	No glaucomatous appearing disc changes (described below), Normal visual field (describe below), statistically normal IOP (<21 mmHg)
Ocular hypertension	No glaucomatous appearing disc changes (described below), Normal visual field, statistically elevated IOP (≥21 mmHg).
Glaucoma suspect	The presence of glaucomatous appearing disc changes (described below), Normal visual field.
Glaucoma	The presence of glaucomatous appearing disc changes (described below) and an abnormal visual field.
Glaucomatous appearing optic disk	Evidence of excavation, neuroretinal rim thinning or notching, localized or diffuse retinal nerve fiber layer (RNFL) defect, or a between eye asymmetry of the vertical cup disc ratio >0.2.
Glaucomatous visual field defect	A reliable standard automated perimetry (SAP) Humphrey 24-2 field (defined as <33 % false positives, false negatives, and fixation losses) that exhibits a pattern standard deviation outside the 95 % normal limits or a glaucoma hemifield test outside of the 99 % normal limits consistent with a RNFL defect pattern based on clinical review.

### New clinic patients

#### Patients diagnosed as normal by the optometrist based on the CEE

**Fig. 1:**
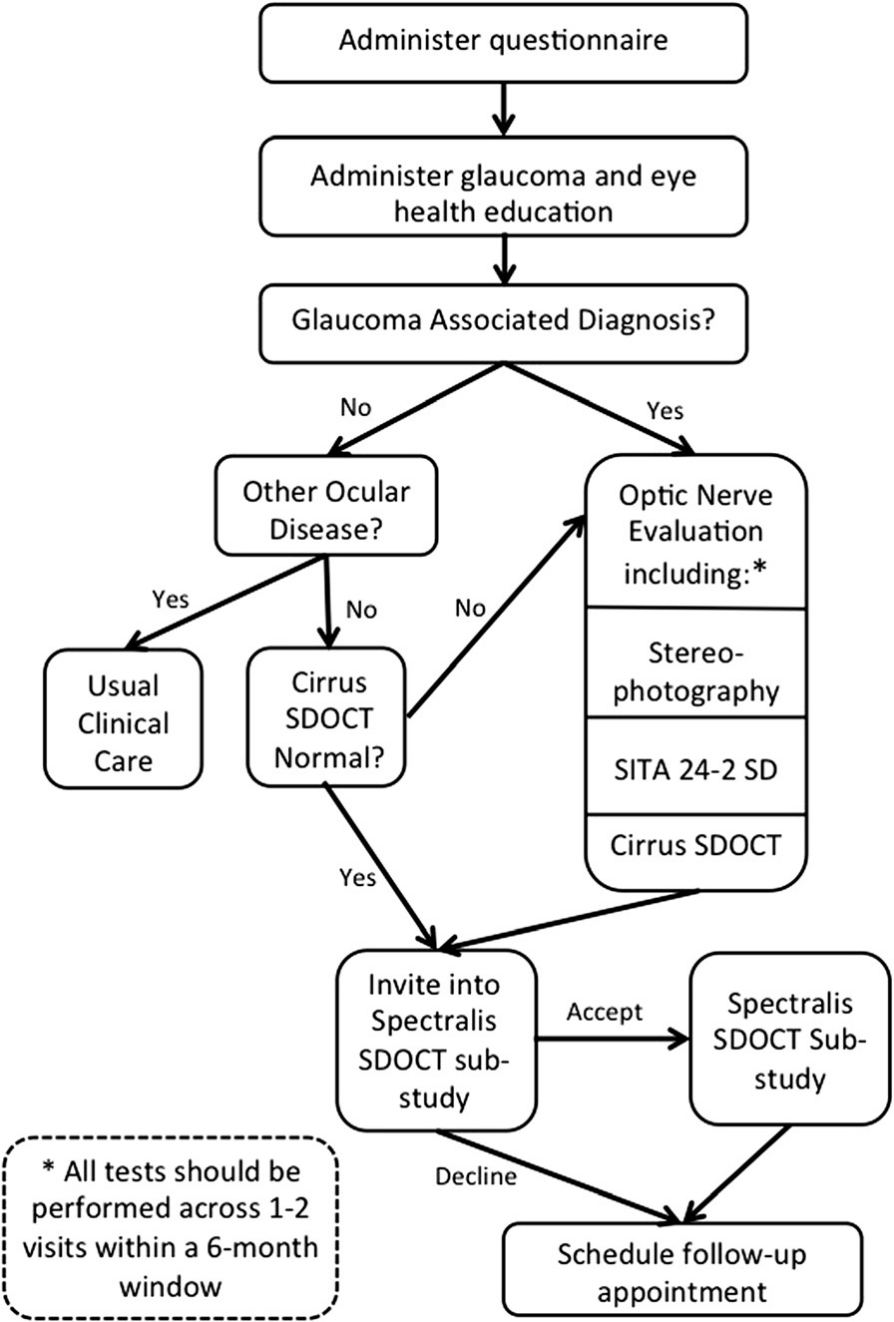
Baseline flowchart for at-risk patients who do not have a GAD diagnosis

Those diagnosed as normal undergo spectral domain optical coherence tomography in both eyes (SDOCT) (Cirrus, Carl Zeiss Meditec,®™ Dublin, CA, USA) (Fig. 1). If the results of the Cirrus SDOCT are normal in both eyes as indicated by a normal retinal nerve fiber layer free of defects, the patient is scheduled for another CEE in 1 year. The results of the Cirrus SDOCT, including the images as well as results from the CEE, are added to the database for remote viewing by the glaucoma specialist at the UAB Glaucoma Service. The patient is then invited to participate in a sub-study using the Spectralis SDOCT (Heidelberg Engineering, Heidelberg, Germany). This sub-study was designed to explore the sensitivity and specificity of a new image analysis approach that has shown promise as a screening method for glaucoma [96].

For patients diagnosed as normal on the CEE but abnormal on the Cirrus SDOCT without a definable cause other than a presumptive GAD, the patient’s diagnosis is converted to GS. Optic nerve head stereoscopic photos are obtained and a follow-up appointment is scheduled within 1 month. This follow-up examination is scheduled in the morning if the initial exam was in the afternoon, and vice-versa, in order to capture diurnal intraocular pressure variations. Standard automated perimetry using the Swedish interactive thresholding algorithm (SITA) (Carl Zeiss Meditec, Dublin, CA, USA) is performed at this visit.

#### Patients diagnosed with a GAD by the optometrist based on the CEE

At the initial exam these patients undergo optic nerve head stereoscopic photography. Either Standard SITA 24-2 perimetry or a Cirrus SDOCT is performed at the initial visit, depending on the optometrist’s decision. The patient is also invited to participate in the Spectralis SDOCT substudy. A follow-up examination is scheduled within 1 month in order to complete the functional and structural assessment of the optic nerve with whichever test, either the Standard SITA 24-2 perimetry or a Cirrus SDOCT, was not completed at the initial visit. This follow-up examination is scheduled in the morning if the initial exam was in the afternoon, and vice-versa.

#### Patients diagnosed with other ocular disease by the optometrist based on the CEE

These patients are managed by the optometrist as he/she would under usual clinical care.

### Existing clinic patients with a GAD diagnosis per the optometrist

**Fig. 2:**
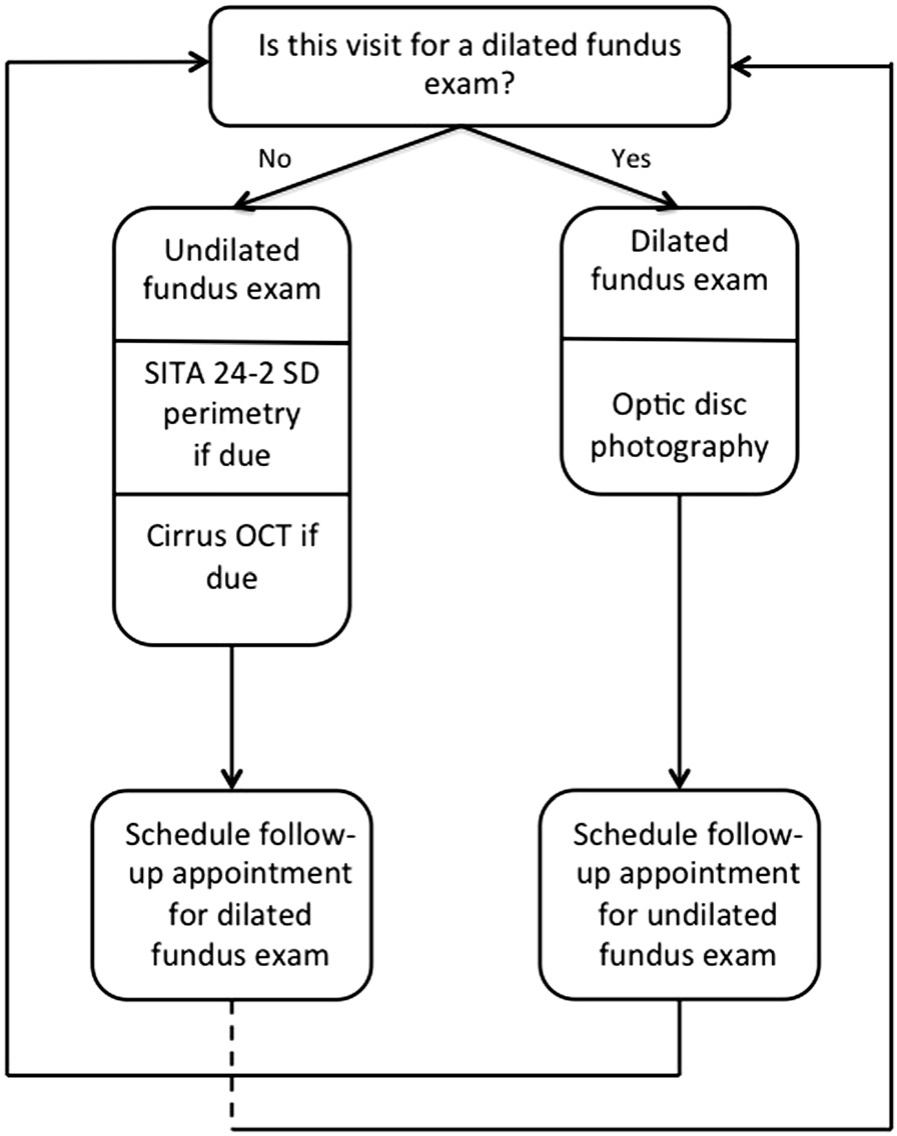
Baseline flowchart for patients who already have a GAD diagnosis

This section pertains to patients who have previously been to the clinic and who have an established GAD diagnosis from the optometrist (Fig. 2). If a dilated fundus exam is scheduled for the patient’s visit, then optic nerve head stereoscopic photography is performed. In addition, the patient is invited to participate in the Spectralis SDOCT substudy. A follow-up examination is scheduled per the optometrist’s recommendation for that patient. The results of these tests are loaded into the electronic database for viewing by the glaucoma specialist located at the UAB Glaucoma Service.

If the visual field and/or SDOCT examination is scheduled for this visit (instead of a dilated fundus examination), then SITA 24-2 standard perimetry and Cirrus SDOCT is performed, if within the billable timeframe. In the US, optic nerve testing such as perimetry, photography, and SDOCT imaging is covered by a patient’s private insurance in general only once a year for each test. If the tests are repeated within a 1-year time period, the patient is often responsible for paying for the cost of the test. Since the goals of this telemedicine program included allowing the optometrist to practice according to his/her usual model of care as well as limiting the patient’s financial burden of glaucoma care, optic nerve testing of existing GAD patients in this study occurred at irregular time periods within the study period. A follow-up examination is scheduled per the optometrist’s recommendation for that patient, generally between 3 and 6 months depending on the severity of the GAD. The results of these tests are loaded into the electronic database for viewing by the glaucoma specialist at the UAB Glaucoma Service.

### Glaucoma reading center

The Glaucoma Reading Center is based at the Glaucoma Service in the Callahan Eye Hospital Clinic of the Department of Ophthalmology at UAB. The database containing the results of the CEE and all additional optic nerve head imaging and functional testing is accessed by a glaucoma specialist in the UAB Glaucoma Service. The glaucoma specialist is initially masked to the optometrist’s diagnosis and treatment plan when electronically viewing the optic nerve head imaging and functional testing as well as CEE for each patient. The glaucoma specialist then enters his/her own diagnostic impressions with respect to GAD and makes recommendations for what treatment (if any) is needed along with frequency of follow-up and interval testing. These recommendations are based within the guidelines of the PPP of the American Academy of Ophthalmology and communicated to the optometrist. Once the glaucoma specialist enters a diagnosis and treatment plan, he/she is able to view the optometrist’s diagnosis and treatment plan in the database. Any disagreement in diagnosis or treatment plan is adjudicated via telephone conversation between the glaucoma specialist in the UAB Glaucoma Service and the optometrist, as occurs with standard consultation referrals. The final treatment plan is communicated to the patient by the optometrist, who continues to manage the patient’s disease within the Vision Center clinic. However, patients deemed by the glaucoma specialist or optometrist’s clinical judgments to have advanced disease in either eye, to be in need of surgical or laser intervention, or to be diagnostically complex are referred to the UAB Glaucoma Service for an onsite evaluation and treatment. In cases of poor data quality (e.g., uninterpretable photos or low reliability indices on perimetry or SDOCT) inadequate to determine a definitive diagnosis, patients are contacted for repeat evaluation and diagnostic testing at the Vision Center, if deemed feasible and necessary based upon telephone communication between the optometrist and glaucoma specialist after joint review of the existing data.

With respect to evaluating the Cirrus SDOCT results at the Glaucoma Reading Center, the following protocol is followed by the glaucoma specialist: (1) Verify patient name, study identification number, and examination date. (2) Review the image quality including whether motion artifact is present, and then identify the signal strength on a scale of 1–10. Poor signal strength is ≤3, moderate signal strength is 4–6, and good signal strength is >7. (3) Review retinal nerve fiber layer (RNFL) deviation map for general rim contour and cup size and identify defects. (4) Review RNFL thickness plot to compare with deviation map. (5) Review extracted horizontal and vertical tomograms for artifacts such as posterior vitreous detachment and drusen. (6) Make a diagnosis, after reviewing the above measures, of Normal, Glaucoma, Suspect, Other/specify. The glaucoma specialist enters all the above judgments into the database.

With respect to evaluating the visual field results at the Glaucoma Reading Center, the following protocol is followed by the glaucoma specialist: (1) Verify patient name, study identification number, examination date and testing strategy (i.e., SITA 24-2 Standard). (2) Review the reliability indices including fixation losses and false positive errors with >33 % of any of the measures considered “unreliable.” (3) Review the pattern standard deviation for areas triggered at 5 % or less. (4) Review the Glaucoma Hemifield Test for deviation from normal limits. (5) Review the Visual Field Index (VFI) for a percentage less than 100 %. (6) Review the Mean Deviation (MD). (7) Make a diagnosis, after reviewing the above measures, of Normal, Glaucoma, Suspect, Other/specify. The ophthalmologist enters all the above judgments into the database.

The following protocol is followed by the glaucoma specialist at the Glaucoma Reading Center to evaluate the stereoscopic fundus photos: (1) Verify patient name, study identification number, and examination date. (2) Determine the image quality for clarity of the optic nerve head as good, moderate, or poor. (3) Examine the optic nerve head for general rim contour and cup size, and identify defects such as notching, rim thinning, or disc hemorrhages. (4) Examine the peripapillary retina for the presence, location and extent of retinal nerve fiber layer defects and beta-zone atrophy. (4) Review retinal vessels and retina for pathology. (5) Make a diagnosis, after reviewing the above measures, of Normal, Glaucoma, Suspect, Other/specify. The ophthalmologist enters all the above judgments into the database.

### Questionnaire administration

The project coordinator administers a questionnaire while the patient is dilating for the CEE at the Vision Center, before the patient receives any results or diagnostic impressions from the optometrist. This questionnaire contains the following item domains (Table 2): (1) eye care utilization [97], (2) trouble seeing, (3) visual task difficulty [98], (4) accessibility/transportation [44], (5) review of chronic medical conditions [99], (6) attitudes about eye care [44], (7) knowledge about glaucoma [43], (8) cost [44], and (9) education completed. The questionnaire responses are not available to the optometrist at any time during the visit.

**Table 2:** Baseline questionnaire, before CEE and eye health education

Domain	Item synopsis
Eye care utilization	When was the last time you had an eye exam in which your pupils were dilated?
Trouble seeing	Do you have any trouble seeing, even when wearing glasses or contact lenses?
	(Yes/no)
Visual task difficulty	Because of your eyesight:
	How much difficulty do you have reading ordinary print in newspapers?
	How much difficulty do you have going down steps, stairs, or curbs in dim light or at night?
	How much difficulty do you have finding something on a crowded shelf?
	(Difficulty scale)^a^
Accessibility/transportation	How much difficulty did you have finding a way to get here? (Difficulty scale)^a^
	I’m more likely to go to the eye doctor if the doctor’s office is near a place I shop
	(Agree/disagree scale)^b^
Review of chronic medical conditions	Have you ever been told by a doctor or other health professional that you have . . .? (Yes/no)
Attitudes about eye care	It is important to go to the eye doctor at least once every 2 years.
	There is no need to go to the eye doctor if you’re not having a problem with your eyes.
	(Agree/disagree scale)^b^
Knowledge about glaucoma	A person can have glaucoma and not know it.
	Glaucoma can be controlled.
	Vision lost from glaucoma can be restored.
	A complete glaucoma exam consists only of measuring eye pressure.
	People at risk for glaucoma should have an eye examination through dilated pupils.
	(True/false)
Cost	Is the cost of an eye exam a problem for you?
	Is the cost of buying eyeglasses a problem for you?
	If the doctor prescribed eye drops for you in order to treat an eye problem you have, would the cost of prescription eye drops be a problem for you?
	(Problem Scale)^c^
Education completed	How many years of school did you complete?

^a^Difficulty scale response options: No difficulty, A little difficulty, Moderate difficulty, Extreme difficulty, Unable to do because of eyesight, Do not do this for other reasons

^b^Agree/disagree scale response options: Strongly agree, Somewhat agree, Somewhat disagree, Strongly disagree

^c^Problem scale response options: Not a problem at all, A little bit of a problem, Somewhat of a problem, A big problem

### Eye health education

The eye health education program administered in this project was specifically designed for the program’s purposes by authors CO, LAR, MB, NP, and CAG. The first education goal is directed at educating the Vision Center clinic staff about how to impart evidence-based information to their patients about GAD, since all patients in the program are at-risk for glaucoma. Staff education occurs before the start of the project. This training is accomplished through the Glaucoma Educator Training Course [100], a free online training program. The program is based on materials available through Prevent Blindness [101] and our previous eye health education program, InCHARGE [23, 44, 40, 50]. The EQUALITY education program provides training in what glaucoma is and how it is treated, persons at risk for glaucoma, how to discuss glaucoma with patients, and how to help patients overcome the barriers to care. The web-based course is followed by an online test to ensure that messages were successfully retained by the trainee. All Vision Center staff members are required to pass the online test, upon which they receive a certificate of completion from Prevent Blindness.

The other goal is to directly educate patients at risk for glaucoma using several approaches. The baseline questionnaire described above is administered before presentation of eye health education in order to ascertain changes in knowledge from before to after the educational information and clinic visit. (1) Videos for patients: Two short videos have been created presenting messages about the importance of routine dilated comprehensive eye examinations for persons who are at-risk for glaucoma. These videos are approximately 3 min long each and are shown to the patient by the project coordinator on an iPad while they are waiting for their pupils to dilate in preparation for the CEE. The videos are available at the Glaucoma Educator Course website [100]. (2) Package “inserts” on glaucoma: These are colorful and to-the-point brochures on who is at risk for glaucoma and the importance of routine eye examination for this at-risk population. The inserts are provided to patients at-risk for glaucoma after the videos are presented, and are also placed in the bags of purchases made by customers in the Walmart Optical Shop and the Walmart Pharmacy in an effort to educate the general public. (3) Posters: These posters (18 × 24 in.) contain material identical to the inserts. They are positioned in the Vision Center, the Walmart Optical Shop, and Walmart Pharmacy.

### Telephone follow-up questionnaire

Approximately 2 to 4 weeks after a patient enters the project, we repeat the administration of some of the questionnaire items by telephone, and add some new items (Table 3). This telephone questionnaire allows for the evaluation of the impact of the EQUALITY program with respect to the following domains: (1) patient satisfaction, (2) other uses of the large retailer adjacent to the Vision Center on the day the patient had an appointment at the Vision Center, (3) accessibility/transportation, (4) eye care utilization and whether prescriptions from the optometrists were filled, (5) attitudes about eye care, (6) knowledge about glaucoma, and (7) cost. Calls are conducted by trained program staff in the UAB Department of Ophthalmology. Up to 10 attempts to contact each patient are made, ensuring that these calls are made at different times of the day in order to facilitate reaching the patient. All questionnaire responses are recorded in the project database, but the responses are not made available to the optometrists or ophthalmologists.

**Table 3:** Follow-up questionnaire administered by telephone 2–4 weeks after the enrollment visit

Domain	Item synopsis
Patient satisfaction	How satisfied were you with your comprehensive eye exam visit?
	(Satisfied/dissatisfied scale)^a^
	How convenient was if for you to have your eye exam at this location?
	(Convenient/inconvenient scale)^b^
	Would you recommend this eye clinic to a friend or family member?
	(Likely scale)^c^
Other uses of large retailer	While you were there for your exam, did you use the Walmart pharmacy?
	Other than the pharmacy, did you do any shopping at Walmart on that day?
	(Yes/no)
Accessibility/transportation	I’m more likely to go to the eye doctor if the doctor’s office is near a place I shop
	(Agree/disagree scale)^d^
Eye care utilization	How likely are you to go for a comprehensive eye exam in the next year or two?
	(Likely scale)^c^
Attitudes about eye care	It is important to go to the eye doctor at least once every 2 years.
	There is no need to go to the eye doctor if you’re not having a problem with your eyes.
	(Agree/disagree scale)^d^
Knowledge about glaucoma	A person can have glaucoma and not know it.
	Glaucoma can be controlled.
	Vision lost from glaucoma can be restored.
	A complete glaucoma exam consists only of measuring eye pressure.
	People at risk for glaucoma should have an eye examination through dilated pupils.
	(True/false)
Cost	Is the cost of an eye exam a problem for you?
	Is the cost of buying eyeglasses a problem for you?
	If the doctor prescribed eye drops for you in order to treat an eye problem you have, would the cost of prescription eye drops be a problem for you?
	(Problem scale)^e^

^a^Satisfied/dissatisfied scale response options: Very satisfied, Satisfied, Dissatisfied, Very Dissatisfied

^b^Convenient/inconvenient scale response options: Very convenient, Convenient, Inconvenient, Very inconvenient

^c^Likely scale response options: Very likely, Somewhat likely, Not very likely, Not at all likely

^d^Agree/disagree scale response options: Strongly agree, Somewhat agree, Somewhat disagree, Strongly disagree

^e^Problem scale response options: Not a problem at all, A little bit of a problem, Somewhat of a problem, A big problem

### Pharmacy data

We are obtaining information on filled prescriptions for all medications prescribed for the treatment of GAD from the Walmart Pharmacies adjacent to the two Walmart Vision Centers. Prescription data will be obtained for the period of June 1, 2012 through August 2014; these dates include an approximate 12 month period before the implementation of the EQUALITY program, and then approximately 12–14 months during the EQUALITY program. We are interested in whether the presence of the EQUALITY program in the Vision Center increased the number of prescriptions for GAD filled in the pharmacy. As a control comparison, we are also obtaining the same type of prescription data from two Walmart Pharmacies adjacent to Walmart Vision Centers where the EQUALITY program was not administered. The prescription information will be obtained for the same time period as mentioned above. The control pharmacies are located in Walmart Supercenters in Bessemer, Alabama, and Fairfield, Alabama, communities with highly similar racial/ethnic and socioeconomic characteristics to the communities served by the Homewood and Tuscaloosa, Alabama Walmart Supercenters. The following pharmacy information will be obtained for medications that are prescribed by doctors for the treatment of glaucoma: patient name and address, date of birth, prescribing doctor, date prescribed, date filled, Walmart pharmacy location, prescription total price, payment by insurance (if applicable), and National Drug Code (NDC). We recognize that not all patients will fill their prescriptions from the optometrist at the Walmart pharmacy; however, many patients do, and thus prescription data could be an important variable to assess.

### Evaluation of research questions

For each of the research questions, we describe the hypotheses to be evaluated, followed by an analytic plan.

1. What is the impact of patient education within study sites about GAD and the importance of routine comprehensive eye care on a patients’ knowledge about GAD and eye care and adherence with recommended follow-up examinations for GAD?
  Hypotheses: (a) Patients’ knowledge about GAD and the importance of routine comprehensive eye care as assessed by our questionnaire at baseline will improve after the EQUALITY program as assessed by the follow-up questionnaire 2–4 weeks after enrollment. (b) Greater improvement in knowledge is associated with greater adherence with (attendance at) follow-up eye examinations. (c) Greater improvement in knowledge is associated with improved adherence to getting GAD prescriptions filled for those who receive GAD prescriptions from the optometrist.
  Analytic plan: Changes in knowledge will be evaluated using statistical tests appropriate for matched samples, specifically McNemar’s test (or the Stuart-Maxwell test) and the paired *t*-test. Correlation coefficients will be used to evaluate the association between changes in knowledge and changes in compliance as well as their association with adherence to GAD prescriptions.
2. What is the level of patient satisfaction with the EQUALITY teleglaucoma program?
  Hypothesis: (a) Over 75 % of patients enrolled in the EQUALITY program will report being satisfied or very satisfied with their eye care at the Vision Center. (b) Patients with a more rural home location, lower socioeconomic status, lesser education and glaucoma knowledge will have higher rates of satisfactions.
  Analytic Plan: A one-sample test will be used to address hypothesis (a) while Pearson correlation coefficients and analysis of variance (or their non-parametric equivalents) will be used to address hypothesis (b).
3. To what extent does the primary care optometrist within the retail eye clinic follow the PPP in managing the care of those at-risk for glaucoma and those already diagnosed with POAG and to what extent do patients follow the PPP recommendations made by the eye care provider?
  Hypothesis: Adherence to PPP for POAG among retail-based primary eye care providers will be significantly lower than recommended guidelines. (a) Provider-related adherence will be lower than that recommended in the PPP guidelines at the monitored telemedicine sites. (b) Patient related factors (e.g. adherence with follow-up, agreeing to dilation) will be lower than that recommended in the PPP guidelines.
  Analytic Plan: One-sample tests suitable for the dependent variable being analyzed (e.g., continuous, categorical) will be used to compare observed to expected provider- and patient-related factors.
4. What is the extent of diagnostic agreement between the primary care optometrist and glaucoma specialist?
  Hypothesis: The EQUALITY program will be more effective in the detection of POAG than the examining primary care optometrist and will equal the performance of glaucoma specialists.
  Analytic Plan: Statistical methods for evaluating agreement will be used to compare diagnostic agreement, specifically the kappa statistic. Statistical significance will be assessed using McNemar’s test and the Stuart-Maxwell test, as deemed appropriate for the measure being evaluated.
5. What is the cost-effectiveness of the EQUALITY telemedicine program compared to usual in-person care by a glaucoma specialist from a limited societal perspective that will consider the direct costs of the competing models and the travel and time costs to patients?
  Hypothesis: The EQUALITY telemedicine program will be more cost-effective compared with standard in-person care by a glaucoma specialist.
  Analytic Plan: Our analysis will examine the incremental costs differences borne by both payer and patient between the telemedicine care delivery model and standard referral-base care, in association with the diagnostic accuracy as measured by the difference in findings by the retail optometrist and the glaucoma specialist. Areas of cost reduction to payers include potential reduction in recommended frequency of visits, testing and/or treatment. The patient costs analysis will include an allowance for patient time and financial expense of travel to both the retail site and to follow up studies with the glaucoma specialist, parking and co-pays to see a subspecialist. Ideally, a cost-effectiveness analysis would consider the incremental cost of delaying the deterioration of vision to a particular end point such as legal blindness. However, given the duration of the study, effects on true progression cannot be evaluated. If significant differences are seen between treatment recommendations, the effects of these differences will be modeled to estimate the incremental cost of delaying the deterioration of vision to a particular end point such as legal blindness.
6. What is the impact of the EQUALITY program in Walmart Vision Centers on the rate of GAD prescriptions being filled in the Walmart Pharmacy, as compared to that rate for two Walmart Pharmacies where the EQUALITY program was not administered?
  Hypothesis: Due to increased patient adherence and improved glaucoma detection: (a) The rate of GAD prescriptions will be significantly higher during the performance period of the EQUALITY project compared to the rate for the immediate preceding year and year after project completion adjusted for variation in clinical volume. (b) The rate of GAD prescriptions will be significantly higher at the EQUALITY performance sites during the project period compared to volume-adjusted comparisons to two Walmart sites with Vision Centers where the EQUALITY program was not deployed.

## Discussion

Glaucoma diagnosis and management is a good candidate for telemedicine because it requires specialized expert review and depends on ocular imaging and other tests that can be electronically transmitted to distant reading centers where tertiary care specialists can consult on patient cases [74–84]. Persons at increased risk for glaucoma [2, 102, 103] are often from populations with low rates of eye care utilization [3, 104, 105], stemming in part from reduced accessibility to eye care because of travel distance to tertiary care centers [23] and inadequate knowledge about the importance of eye care and common eye diseases [46, 44]. Teleglaucoma programs have the potential to reach these segments of the population, while also potentially reducing the cost of eye care [106, 90]. Eye health education can be built into interactions between the clinician/clinic staff and the patient with the goal of facilitating adherence to attending recommended and routine eye appointments and improving medication adherence [107]. In the US there are several national general-merchandise retailers with eye clinics staffed by a primary care optometrist. For example, Walmart has approximately 3200 Vision Centers distributed throughout the US. The existence of a network of retail-based clinics will facilitate the scalability of the program nationally, should its efficacy, patient acceptance, and cost-effectiveness be demonstrated. In addition, these retailers are typically located within communities where residents frequently visit them for a variety of daily and household needs. Thus, retail-based teleglaucoma programs have the potential to enhance accessibility.

This project is evaluating the feasibility, efficacy, patient acceptance, and cost-effectiveness of a teleglaucoma program that is a novel implementation of telemedicine. In reporting our evaluation of the program in future papers, we will also summarize the challenges faced in administering the program, as well as solutions implemented and lessons learned. As healthcare delivery systems in the US strive to improve quality while reducing costs, telemedicine programs, including teleglaucoma initiatives such as EQUALITY, could contribute toward reaching this goal.

## Abbreviations

CEE: Comprehensive eye examination
EQUALITY: Eye Care Quality and Accessibility Improvement in the Community
GAD: Glaucoma associated diagnosis
GS: Glaucoma suspect
IOP: Intraocular pressure
MD: Mean deviation
NDC: National Drug Code
OHT: Ocular hypertension
POAG: Primary open angle glaucoma
PPP: Preferred practice patterns
RNFL: Retinal nerve fiber layer
SAP: Standard automated perimetry
SDOCT: Spectral domain optical coherence tomography
SITA: Swedish interactive thresholding algorithm
VFI: Visual field index
